# Effects of Repetitive Peripheral Magnetic Stimulation on Hemodynamics Compared to Neuromuscular Electrical Stimulation

**DOI:** 10.1298/ptr.25-E10386

**Published:** 2026-03-07

**Authors:** Takeru ECHIZEN, Shoki YUSU, Urara CHIBA, Tomoyuki MORISAWA, Masakazu SAITOH, Kotaro IWATSU, Tetsuya TAKAHASHI

**Affiliations:** 1 Department of Physical Therapy, Graduate School of Health Science, Juntendo University, Japan; 2 Department of Physical Therapy, Faculty of Health Science, Juntendo University, Japan

**Keywords:** Repetitive peripheral magnetic stimulation, Neuromuscular electrical stimulation, Hemodynamics, Skeletal muscle, Healthy participants

## Abstract

**Objectives:**

This study aimed to compare the effects of repetitive peripheral magnetic stimulation (rPMS) and neuromuscular electrical stimulation (NMES) on hemodynamics and obtain basic data to verify the safety of rPMS in healthy adults.

**Methods:**

rPMS and NMES were performed for 5 min in the supine position using a maximum-intensity stimulation that did not cause pain in the quadriceps femoris of 20 healthy adult males. Regarding the measurement items, the maximum inferior vena cava diameter (IVCDmax) was measured using an echocardiograph to determine the increase or decrease in venous return. Right atrial pressure (RAP) was estimated from the measured IVCDmax and respiratory collapsibility index to determine the cardiac preload. Systolic blood pressure (SBP) and heart rate (HR) were measured as indicators of cardiovascular response.

**Results:**

A significant interaction was observed for IVCDmax (p <0.05). Multiple comparison tests showed no significant change in IVCDmax for NMES but showed a significant increase during stimulation for rPMS (p <0.01). No significant changes were observed in the HR, SBP, or RAP throughout the experiment.

**Conclusions:**

rPMS may produce a stronger muscle pump action than NMES, resulting in increased venous return and IVCDmax expansion. However, since rPMS did not significantly change HR, SBP, or RAP, it is suggested that it does not cause excessive hemodynamic stress in healthy participants.

## Introduction

Neuromuscular electrical stimulation (NMES) has been used to prevent and improve muscle weakness in patients with critical illnesses and cardiovascular diseases^1–3)^. Recently, NMES has been recommended as an alternative to exercise therapy for the prevention and improvement of muscle weakness in patients admitted to the intensive care unit (ICU)^4)^. However, previous studies have reported that NMES stimulation had to be interrupted in some patients due to pain^5)^. In addition, patients with edema require very high stimulation intensity settings to obtain sufficient muscle contraction^6)^. High-intensity stimulation is likely to induce pain. Therefore, patients with unstable hemodynamics who are prone to edema, as well as patients with acute cardiovascular disease, are particularly susceptible to the pain caused by stimulation and are unlikely to obtain sufficient muscle contraction through NMES.

In recent years, repetitive peripheral magnetic stimulation (rPMS) has attracted attention as a new physical therapy for the prevention and improvement of muscle weakness. rPMS induces electrical currents within biological tissues via a time-varying magnetic field generated by an external coil, thereby stimulating motor nerves in a noninvasive manner. Because the induced current is minimally affected by skin impedance, rPMS can activate deeper motor nerve fibers while reducing stimulation of cutaneous nociceptors^7)^. As a result, rPMS has been reported to produce less pain and discomfort and to elicit stronger muscle contractions compared with NMES^8,9)^. Due to these advantages, rPMS is thought to be able to induce sufficient muscle contraction to prevent and improve muscle weakness in patients with edema. Therefore, it is hoped that rPMS will be applied in the future to patients who have difficulty with active mobilization and voluntary exercise, such as patients with acute cardiovascular disease and patients who are critically ill.

However, there have been no reports of rPMS use in patients who are critically ill or those with acute cardiovascular disease. When using rPMS in these patients, particular consideration should be given to its effects on hemodynamics. Notably, there are no reports that have examined the effects of rPMS on hemodynamics in detail, including in healthy adults. In an NMES study in patients with acute myocardial infarction, blood pressure (BP) and heart rate (HR) increased slightly but significantly during and after stimulation^10)^. In addition, previous studies have shown that NMES increases venous return from the lower limbs due to the muscle pump action^11)^. Since rPMS produces stronger muscle contractions than NMES, it may excessively change the HR and BP and increase cardiac preload. To date, there have been no reports of serious adverse events or side effects caused by rPMS^12–16)^; however, if it can be verified that rPMS can be performed without causing excessive changes in hemodynamics, it may be possible to apply it widely in patients with acute cardiovascular disease and those who are critically ill in the future.

Therefore, in this study, we examined the effects of rPMS on hemodynamics in healthy adults to obtain basic data for the safe implementation of rPMS in patients with acute cardiovascular disease and those who are critically ill. In addition, since rPMS causes stronger muscle contractions than NMES, we aimed to examine the differences in the hemodynamic effects between the 2 interventions.

## Methods

### Participants

Twenty healthy young adult males (mean age: 22.7 ± 2.3 years; mean body mass index: 22.9 ± 2.3 kg/m^2^) volunteered to participate in this study. We excluded participants with regular exercise habits at the athlete level, a history of smoking, or respiratory, circulatory, neurological, or orthopedic disorders that could affect exercise function. The study protocol was approved by the Ethics Committee of the Faculty of Health Science, Juntendo University (Approval No. 23-058) and conformed to the principles of the Declaration of Helsinki. Before participating in this study, all participants received a detailed explanation of the experimental protocol and provided written informed consent.

### Study design

**Fig. 1.**
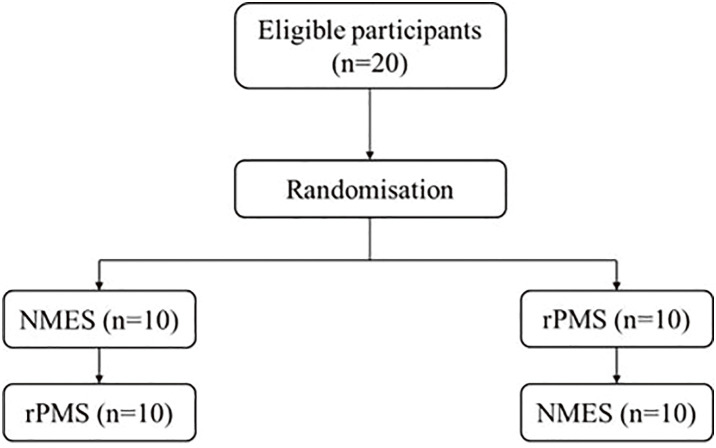
Flowchart of the study. Twenty eligible participants were randomly allocated to 1 of 2 intervention sequences in a randomized crossover design. Participants first underwent either NMES or rPMS, followed by the alternate intervention after a washout period of at least 1 week. NMES, neuromuscular electrical stimulation; rPMS, repetitive peripheral magnetic stimulation

Figure 1 shows the flowchart for this study. This study was a non-blind randomized crossover trial, and participants were assigned to the order of the NMES or rPMS sessions using block randomization to prevent imbalance in intervention order. Allocation was based on a random number table generated using the randomization function of Microsoft Excel by an independent researcher, with intervention sequences balanced within each block. Allocation results were concealed until the start of the first session. Both sessions were performed at intervals of at least 1 week. To minimize the influence of factors affecting hemodynamics and autonomic nervous system activity^17)^, participants were instructed to refrain from vigorous exercise and avoid alcohol and caffeine intake from 1 d before the start of the study.

### Experimental procedure

**Fig. 2.**
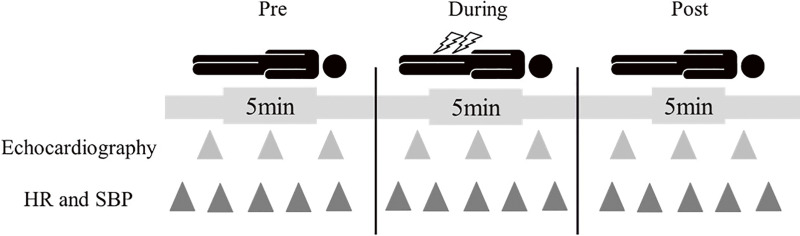
Experimental procedure. Each session consisted of 3 phases: a 5-min resting period before stimulation (pre), a 5-min stimulation period involving NMES or rPMS (during), and a 5-min resting period after stimulation (post). Echocardiographic measurements of the diameter of the inferior vena cava were obtained at predefined time points in each phase, while HR and SBP were repeatedly measured throughout the session. Triangles indicate predefined measurement time points for echocardiography and vital signs. HR, heart rate; SBP, systolic blood pressure; pre, before the NMES or rPMS session; during, during the NMES or rPMS session; post, after the NMES or rPMS session; NMES, neuromuscular electrical stimulation; rPMS, repetitive peripheral magnetic stimulation

Figure 2 shows the experimental procedure for this study. All procedures were performed by the authors, who are registered physical therapists. To minimize the effects of postprandial hemodynamic changes, the experiments were performed at least 2 h after eating, and the participants first rested in the supine position for 5 min (pre-stimulation). After determining the stimulation intensity for rPMS and NMES, NMES or rPMS was performed (during-stimulation) for 5 min. After the stimulation, the participant was again placed in the supine position and rested for 5 min (post-stimulation). This study aimed to evaluate the acute effects of short-duration stimulation on hemodynamics safely, as an initial step in a phased research program. Previous studies have reported acute muscle swelling around the stimulation site, as well as changes in peripheral circulatory responses, even with relatively short rPMS sessions lasting approximately 3 min^14)^. Therefore, this study set the stimulation duration to 5 min. During the experiment, the degree of pain was checked using the Numerical Rating Scale^18)^.

### Measurement of HR and BP

Systolic BP (SBP) and HR were measured as indicators of cardiovascular response to NMES and rPMS. SBP was measured using an automatic electronic blood pressure monitor (Tango M2; SunTech Medical, NC, USA). The HR was recorded using a cardiopulmonary exercise load monitoring device (MLX-1000-CPX; FUKUDA DENSHI, Tokyo, Japan).

### Measurement of maximum inferior vena cava diameter and estimated right atrial pressure

We measured the maximum inferior vena cava diameter (IVCDmax) and estimated the right atrial pressure (RAP) as indicators of venous return and cardiac preload. IVCDmax and RAP were measured based on the 2021 revised guidelines for the indications and interpretation of cardiovascular ultrasound examinations^19)^. First, IVCDmax was observed by drawing a long-axis cross-section using the parasternal long-axis view with an echocardiograph (ACUSON Juniper; Siemens Healthineers, Bayern, Germany). IVCDmax was measured in the immediate vicinity of the confluence of the hepatic veins, which is located at 0.5–3.0 cm from the right atrial inlet^20)^, and it was standardized to measure the time of the end-expiratory phase when the size of the inferior vena cava is at its maximum. The IVCDmax cardiac ultrasound image was measured in the B-mode^21)^.

RAP can be estimated using IVCDmax and the respiratory collapsibility index (CI). In this study, the ratio of the maximum diameter during expiration to the minimum diameter during inspiration (maximum diameter – minimum diameter/maximum diameter) × 100 (%) was used as the CI using the sniff method. If IVCDmax was ≤21 mm and the CI was ≥50%, it was classified as 3 mm Hg. If IVCDmax was >21 mm and the CI was <50%, it was classified as 15 mm Hg. If these findings did not match, they were classified as 8 mmHg^20)^.

In addition, previous studies have reported a slight degree of variation in IVCDmax measurements within the same examiner^22)^. In this study, all IVCDmax measurements were performed by a physical therapist who had practiced the measurement method for over 6 months. In all the study participants, the IVCDmax at rest was measured 3 times before the start of the first experiment, and intraclass correlation coefficients (ICCs) were calculated. The ICC (1, 1) was 0.967 (0.933–0.986, 95% confidence interval), confirming the high reproducibility of the measurement method within the same examiner.

### NMES session

The NMES device (ESPURGE; Ito, Tokyo, Japan) used adhesive electrodes measuring 5 cm × 9 cm. The proximal electrode was placed approximately two-thirds of the way along the lateral border of the rectus femoris muscle, measured from the upper edge of the patella. In contrast, the distal electrode was placed on the belly of the rectus femoris and vastus medialis muscles, approximately 10 cm from the upper edge of the patella^9)^. The stimulation parameters were as follows: frequency, 50 Hz; duty cycle, 1:2 (stimulation time, 3 s; rest time, 6 s); phase duration, 300 µs. The stimulation intensity was defined as the maximum intensity that did not cause pain^10,23)^. Because the dominant leg is more frequently used in daily activities and may exhibit greater variability in neuromuscular function, the non-dominant leg was selected to minimize these potential influences.

### rPMS sessions

rPMS was performed using a round coil with a diameter of approximately 18 cm, attached to the main device (Magrex; MR, Gyeonggi-do, Korea) and placed on the belly of the rectus femoris muscle at a point midway between the upper edge of the patella and the anterior superior iliac spine^9)^. The stimulation parameters were a frequency of 50 Hz and a duty cycle of 1:2 (stimulation time, 3 s; rest time, 6 s). The stimulation intensity was set at the maximum intensity that did not cause pain. Similarly to NMES, rPMS was applied to the quadriceps muscle of the participant’s non-dominant leg. The maximum intensity level of the rPMS device used in this experiment has a maximum output of 7.5 tesla (100%), which is higher than that of rPMS devices commonly used in previous studies (approximately 0.9–3.1 tesla)^9,12–16)^.

### Statistical analysis

The sample size was determined based on an effect size derived from our preliminary data (n = 5). The effect size was 0.297, and the required sample size was calculated using G*Power (version 3.1) for a 2-way repeated-measures analysis of variance (ANOVA) (intervention × time), assuming an α-error of 0.05 and a power of 0.80. The analysis indicated that 20 participants were required; therefore, 20 participants were included in this study. HR and SBP were measured every minute at each time point (pre-stimulation, during stimulation, and post-stimulation), and the mean and standard deviation (SD) were calculated. IVCDmax and RAP were also measured thrice at each time point (pre-stimulation, during stimulation, and post-stimulation), and the mean, SD, and median (25th and 75th percentiles) were calculated for each.

The normality of HR, SBP, IVCDmax, and RAP was tested using the Shapiro–Wilk test. A repeated-measures 2-way ANOVA was conducted within the time factor (pre-stimulation, during stimulation, and post-stimulation) for each intervention group (rPMS and NMES) to compare the changes in HR, SBP, and IVCDmax. When a significant interaction was observed, multiple comparison tests were performed using the Bonferroni method. In addition, changes in RAP were compared using the Friedman and Wilcoxon signed-rank tests (with Bonferroni correction). All analyses were performed using the Statistical Package for the Social Sciences (SPSS version 29.0; IBM Japan, Tokyo, Japan), and p <0.05 was considered statistically significant.

## Results

For both sessions, all participants completed the experiment without serious adverse events or side effects due to the stimulation were observed, and no participant dropped out of this study. In addition, visible muscle contractions caused by the stimulation were induced in the quadriceps muscles of the participants in all sessions of this study. The average stimulation intensity achieved by the participants was 13.2 mA (8–25 mA) in the NMES condition and 22.7% (17%–37%) in the rPMS condition.

### Changes in HR and SBP

No significant interactions or main effects were observed for HR and SBP (Table 1).

**Table 1.** Changes in HR and SBP

		Pre	During	Post
HR (b.p.m.)	NMES	57.5 (±6.2)	57.2 (±6.0)	56.9 (±5.9)
	rPMS	58.8 (±8.7)	58.9 (±9.1)	59.1 (±9.6)
SBP (mmHg)	NMES	114.7 (±9.4)	114.4 (±9.9)	114.8 (±9.4)
	rPMS	115.0 (±9.9)	115.9 (±10.5)	115.2 (±10.4)

Data are presented as mean values (±SD).

HR, heart rate; SBP, systolic blood pressure; NMES, neuromuscular electrical stimulation; rPMS, repetitive peripheral magnetic stimulation; SD, standard deviation; pre, before the NMES or rPMS session; during, during the NMES or rPMS session; post, after the NMES or rPMS session

### Changes in the IVCDmax and estimated RAP

**Fig. 3.**
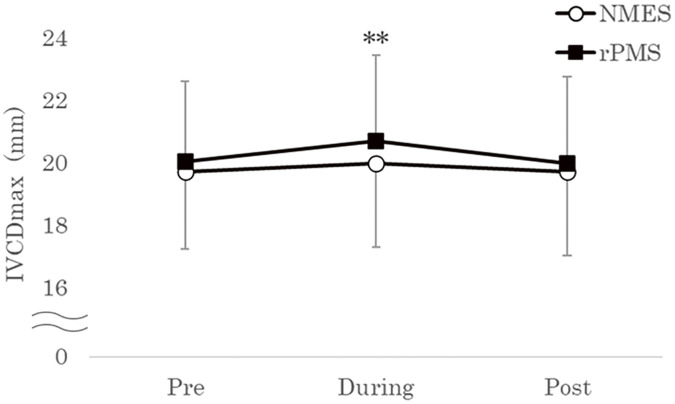
Changes in IVCDmax. The mean changes in IVCDmax pre-, during, and post-stimulation are shown for the NMES and rPMS conditions. Data are presented as mean ± standard deviation. A significant increase in IVCDmax was observed during rPMS compared with the pre-stimulation phase. IVCDmax, maximum inferior vena cava diameter; NMES, neuromuscular electrical stimulation; rPMS, repetitive peripheral magnetic stimulation; pre, before NMES or rPMS session; during, during the NMES or rPMS session; post, after NMES or rPMS session. ^**^p <0.01, compared with before the rPMS session

A significant interaction was observed between the intervention group and time in the IVCDmax (F [2, 38] = 4.106, p <0.05, η^2^ = 0.178). Multiple comparison testing showed no significant change in IVCDmax throughout the experiment in the NMES group (pre: 19.7 ± 2.5 mm vs. during: 20.0 ± 2.7 mm vs. post: 19.7 ± 2.7 mm) (Fig. 3). However, in the rPMS group, the IVCDmax during stimulation was significantly higher than that during pre-stimulation (pre: 20.0 ± 2.6 mm vs. during: 20.7 ± 2.8 mm, 95% CI = +0.377 to +0.943, p <0.01) (Fig. 3).

No significant main effects were observed in RAP between the intervention groups or time periods (Table 2).

**Table 2.** Changes in RAP

		Pre	During	Post
RAP	NMES	3 (3, 8)	3 (3, 8)	3 (3, 8)
(mmHg)	rPMS	3 (3, 8)	3 (3, 8)	3 (3, 8)

Data are presented as medians (25th and 75th percentiles).

RAP, right atrial pressure; NMES, neuromuscular electrical stimulation; rPMS, repetitive peripheral magnetic stimulation; pre, before NMES or rPMS session; during, during NMES or rPMS session; post, after NMES or rPMS session

## Discussion

In this study, we examined the effects of rPMS on the cardiovascular response and cardiac preload indices in healthy adult males and compared them with those of NMES. The results showed that neither intervention groups caused significant changes in HR or SBP. In contrast, the rPMS group showed significantly higher IVCDmax values during stimulation; however, no significant changes were observed in RAP throughout the experiment. Therefore, this is the first study to show that rPMS can be performed without inducing excessive cardiovascular responses or excessively increasing the cardiac preload in the same manner as NMES.

Regarding changes in cardiovascular responses, previous reports showed that NMES can be performed in healthy adults, patients with acute cardiovascular disease, and those who are critically ill without causing excessive changes in HR or SBP^10,23–26)^. In these studies, stimulation intensities in the “no pain” or “no excessive pain” range were used. The relatively low-intensity stimulation was used because excessive activation of the sympathetic nervous system by painful stimulation may adversely affect cardiovascular responses. Excessive sympathetic nervous system activation can lead to the development of arrhythmias, excessive increases in BP and HR, and even sudden death^27)^. In this study, the stimulation intensity of NMES and rPMS was set to the “maximum intensity within the no pain range,” and the experiment was completed without inducing excessive changes in HR or SBP. Therefore, we believe that the stimulation parameters of NMES and rPMS used in this study can be implemented without negatively impacting the cardiovascular responses.

In this study, no significant changes were observed in IVCDmax in response to NMES. Hoshiai et al.^28)^ reported that no significant changes were observed in IVCD in response to NMES in healthy adult males. Therefore, the results of the present study support those of previous studies. In contrast, in this study, IVCDmax was significantly higher during the rPMS session. This finding is likely attributable to the specific stimulation characteristics of rPMS. Unlike NMES, rPMS induces muscle contractions with less discomfort, allowing stimulation to be applied at higher intensities without pain. Indeed, Han et al.^9)^ reported that rPMS elicits stronger quadriceps muscle contractions than NMES under pain-free conditions. In addition, Struppler et al.^7)^ reported that rPMS can activate deeper motor nerve fibers. Based on these characteristics, it is reasonable to assume that rPMS induced relatively greater muscle contraction in the present study, resulting in enhanced muscle pump activity in the lower limb. Increased muscle pump action is known to augment venous return, which likely contributed to the observed increase in IVCDmax during rPMS.

However, in critically ill patients with acute cardiovascular disease, increases in venous return induced by muscle contractions may excessively elevate cardiac preload and destabilize hemodynamics, making a safety assessment essential. Kondo et al.^25)^ reported that, in a study on NMES in patients with acute heart failure, there were no significant changes in RAP before or after stimulation, and no patients experienced a worsening of their heart failure symptoms.

In this study, IVCDmax increased significantly during rPMS; however, the magnitude of change was small, and no significant changes were observed in estimated RAP. Because RAP reflects cardiac preload, the absence of changes in RAP suggests that the increase in venous return induced by rPMS did not translate into excessive preload elevation. Therefore, it is thought that the stimulation parameters used in this study had a slight effect on cardiac preload. In the future, the safety and efficacy of rPMS warrant consideration as an adjunctive intervention for critically ill patients and those with acute cardiovascular disease who require hemodynamic stability.

This study has several limitations. First, all the study participants were healthy adult males. Further verification is required to determine whether rPMS can be widely applied as an alternative to exercise therapy, considering factors such as gender, age, and disease. Second, this study did not directly assess cardiac output or stroke volume. A quantitative echocardiographic evaluation of these parameters requires precise measurement of the diameter of the left ventricular outflow tract and the velocity–time integral derived from Doppler ultrasound, as well as strict control of measurement reproducibility. Due to the measurement environment and examination framework of this study, it was considered difficult to reliably and consistently acquire these parameters from all participants. Third, we considered that the difference in muscle contraction force between NMES and rPMS was related to the mechanism of the increase in IVCDmax observed in this study. However, in this study, it was difficult to quantitatively evaluate muscle contraction force during the NMES and rPMS sessions using electronic devices to avoid interference with peripheral devices. Therefore, in future studies, it is necessary to verify the muscle contraction force actually induced by the stimulation parameters of rPMS used in this study using an appropriate evaluation method. Fourth, it is unclear whether the stimulation parameters used in this study actually prevent or improve muscle weakness. In this study, the stimulation intensity of rPMS was set based on NMES studies targeting patients with acute cardiovascular disease or critically ill patients. It will be valuable to consider immediate and long-term effects in the future to determine whether rPMS is effective for those patients.

## Conclusions

Our findings showed that the rPMS stimulation parameters used in this study can be implemented in healthy adult males without inducing excessive cardiovascular responses or cardiac preload.
